# A qualitative assessment of medical students’ readiness for virtual clerkships at a Qatari university during the COVID-19 pandemic

**DOI:** 10.1186/s12909-023-04117-3

**Published:** 2023-03-27

**Authors:** Hiba Bawadi, Ayad Al-Moslih, Rula Shami, Xiangyun Du, Alla El-Awaisi, Hanan Abdul Rahim, Ghadir Fakhri Al-Jayyousi

**Affiliations:** 1 Section Head of Clinical Education, QU Health, Vice President for Medical and Health Sciences Office, Qatar University, PO Box 2713, Doha, Qatar; 2 Section Head of Pre-Clinical Education, College of Medicine, QU Health, Qatar University, PO Box 2713, Doha, Qatar; 3 College of Health Sciences, QU Health, Qatar University, PO Box 2713, Doha, Qatar; 4 College of Education, Qatar University, PO Box 2713, Doha, Qatar; 5 College of Pharmacy, QU Health, Qatar University, PO Box 2713, Doha, Qatar

**Keywords:** Medical students, Readiness to emergency change, Virtual clerkship, Health education, COVID-19, Qatar

## Abstract

**Background:**

This study aims to qualitatively examine the readiness of medical students to change to virtual clerkship (VC) during the pandemic, from both the faculty and students’ perspectives.

**Methods:**

A qualitative study was conducted based on the framework of readiness to change. Focus group discussions with students, and semi-structured interviews with clinical faculty members were done using appropriate online platforms. Transcripts were then analyzed using inductive-deductive approach.

**Results:**

Twelve themes emerged which are (1) Perceptions about the university’s decision and its communication to students, (2) A Perceived lack of clinical experience, (3) Students’ role as members of the medical team facing the pandemic, (4) Student safety, (5) Quality and design of VC and the skills it offered, (6) Belief in own ability to succeed in the VC, (7) Confidence that VC would reach its goals, (8) New enhanced learning approaches, (9) Preparing students for new types of practice in the future (10) Acquired skills, 11) Academic support and communication with faculty and college, and 12) Psychological support. Medical students showed limited readiness to undertake a virtual clerkship and not play their role as healthcare professionals during the pandemic. They perceived a huge gap in gaining clinical skills virtually and asked for a quick return to training sites.

**Conclusion:**

Medical students were not ready for virtual clerkships. There will be a need to integrate novel learning modalities such as patient simulations and case-based learning in order to meet future demands of the medical profession and enhance the efficiency of virtual clerkships.

**Supplementary Information:**

The online version contains supplementary material available at 10.1186/s12909-023-04117-3.

## Background

In 2020, the COVID-19 pandemic resulted in a sudden disruption to education worldwide. It had a deep impact on both medical practice and medical education. Its expansion coincided with the start of the second half of the academic year in most academic institutions, a time when medical students and other health professions students had only completed a fraction of their clinical training, and hence had only partially acquired the essential clinical competencies they were supposed to gain as the main learning outcome of their training. Regular classes were shifted to online lectures and case-based discussions, and hospitals had to prioritize pandemic preparedness. Hence, medical academic programs struggled with finding a solution to avoid compromising the quality of experiential learning that had to be delivered to medical students during that time. They were also trying to balance the adequate level of teaching social and professional responsibilities and ensuring the physical and psychological well-being of medical students.

Qatar University Health cluster (QU Health) constitutes of the Colleges of Medicine (CMED), Health Sciences, Pharmacy, and Dental Medicine at Qatar University (QU); the national university in the state of Qatar. The Doctor of Medicine (MD) program at CMED is six years in length. The program adopts the six domains of competence described by the Accreditation Council of Graduate Medical Education, ACGME [1]. After finishing a foundation year, MD students complete a pre-clerkship for two and a half years then a clerkship training for another two and a half years. The latter starts with an introductory clerkship training over a period of 14 weeks where the students spend half of it rotating between general medicine including wards, outpatient clinics and attend relevant diagnostic or therapeutic procedures, and spend the other half similarly however in general surgical care services [2].

During the COVID-19 pandemic, and like many similar programs around the world, the MD program at CMED was faced with a need for creating safe and appropriate alternatives for onsite clinical training. Thus, clerkships were shifted to online platforms with emphasis on delivery of clinical knowledge and reasoning through means of case discussions. Such online sessions were delivered via platforms which are supported by QU like Cisco Webex and Microsoft Teams. This shift was guided by evidence from literature about the benefits of introducing interactive internet-based learning to medical curricula across different healthcare-related fields, [3–6]. The MD program at CMED divided the time of the clerkship between online lectures and study sessions, while it deferred clinical placements until after the restrictions of clinical attachments were lifted in July 2020. Meanwhile, the program ensured students were educated about the required clinical knowledge for their clinical placements. This time was also targeted for the completion of in-program assessments such as case reports, case logs, case presentations and work-based assessments. The assessment involved case discussions. Students were asked questions about the history and physical examination findings of certain medical conditions. Questions covered medical conditions that patients would commonly present with. An assessment blueprint ensured fair distribution of questions across the curriculum content. Assessment also targeted clinical reasoning, diagnosis, investigation, and treatment across a range of clinical problems. Case discussions were conducted online, but subsequent case discussions were run in the multiple mini interview formats in house. The missing element was performance-based student assessment which usually involved patients encounters such as the Objective-Structured Clinical Exam (OSCE) and workplace-based assessment.

Implementing virtual clerkships VCs demands major changes at various levels. Research on change management has consistently stressed the importance of readiness as a main driver for embracing change [7]. Earlier literature on change management proposed some models for readiness of change. A model constructed by Armenakis et al. [8, 9] suggests five key areas that need to be tackled to communicate and facilitate the change: discrepancy, appropriateness, efficacy, principal support, and valence. This framework of readiness can be applied to understand CMED student readiness for shifting to VC in a context of COVID-19 emergency. By applying this model, these aforementioned five key areas should be explored upon addressing the change message to medical students and faculty.

A number of studies had generally addressed medical students’ perspectives for doing their internships virtually during the Covid-19 pandemic [10–14]. However, the majority of published literature on readiness to online education were done in normal situations [3, 4, 15, 16] and may not be able to describe precisely how medical students’ readiness to change will look like in the presence of a pandemic; a time when change happened abruptly and was by no means a mere choice. Many of the available studies were done to assess medical student perceptions in the context of blended internships [17, 18].Thus, there is also scarcity in literature about medical students’ readiness to transition to emergency full-scale VC, as happened in the Spring 2020 academic semester. It would therefore be crucial to understand medical students’ initial readiness and experiences in such situations in order to ensure better clerkship outcomes. It would also be important to explore how a readiness framework would apply in the context of the externally enforced decision evoked by the Covid-19 pandemic. Hence, the purpose of this qualitative research is to study the readiness of CMED students at QU to change to VC, from their own perspective as well as from the QU clinical faculty perspectives.

## Methods

### Study design

This qualitative study is part of a large project that aimed to explore the experience of switching to virtual internships during the pandemic from both the clinical instructors’ and students’ perspectives at QU Health [19, 20]. The study has adopted the concepts of readiness for change in an attempt to understand how clinical faculty members and CMED students at QU perceived students’ readiness to the shift to VC during the COVID-19 pandemic. Using an inductive-deductive approach, we aimed to gain understanding of students’ experiences during this shift.

The research team was able to develop an interview guide and a focus group guide considering the main constructs of the readiness framework and by reviewing the relevant literature and discussing its findings among themselves. The study took place in May which is near the end of the spring 2020 term, around the time when the Covid-19 pandemic was at its peak. It ran in two parallel streams within CMED: focus groups with students and semi-structured, one-to-one interviews with clinical faculty members.

Focus groups involved clerkship students of different rotations and academic levels from years four and five. As the student focus groups were mixed years, the moderator ensured to indicate as part of the ground rules stated in the beginning that participants are encouraged to follow the sequence of names as seen listed on the Webex platform which was used for these focus group discussions. Students took the chance to speak when they noticed a few seconds pause or silence by the person whose name preceded them on the list. The moderator checked again if all students shared what they wanted to say, before moving to the next question. In addition five semi-structured one-to-one interviews with QU clinical faculty members. Both streams were run by a trained research team member who was not involved in coordinating nor delivering clinical training for the students, and utilized WebEx and Microsoft-teams online platforms. Another member of the research team was also responsible for taking detailed written notes to serve as a reference in addition to the transcribed audio recordings.

#### First stream: students

An email that included a description of the study and an invitation to participate through an online platform was sent out to all fourth and fifth year CMED students (N = 111) who were registered for clinical training, only 11 consented to participate.

The invitation also included a consent form, which students were asked to sign before joining the focus group. Students who returned a signed consent form were then emailed regarding the planned date and time of the focus group and received a calendar invitation and a link to the electronic platform. At the beginning of the focus group discussion, the focus group facilitator reiterated the contents of the consent form and explained the rules of conduct in the group ahead of the start of the focus group discussion. Each focus group involved five to six clerkship students of different rotations and academic levels from years four and five. After conducting the first focus group, more probing questions were added to the discussion guide to elicit rich information and encourage students to elaborate on their answers in the second focus group where data saturation was reached. Researchers reach data saturation when they feel they are not gaining any new insight and that they can stop the data collection [21]. The focus groups, conducted in English, were conducted online, each lasting 60–70 min. No incentives were offered to students who participated.

#### Second stream: clinical faculty members

Similarly, clinical faculty members from CMED were contacted via an email, which included a description of the study and an invitation to participate in an interview through an online platform. Ten faculty members from both the medical and surgical rotations were invited, however only five consented and were available for an interview. Faculty members were also asked for permission to record the session, each lasting 45 min.

Both the focus groups and interviews were conducted in English since all participants were fluent in English. The consent form and all study protocols were approved by the Institutional Review Board at Qatar University (QUERG-CHS-2020-1). All methods were carried out in accordance with relevant guidelines and regulations. A signed informed consent form was obtained from all participants by email.

### Data analysis

Students’ focus group discussions were audio recorded and transcribed verbatim. We used the constant comparative method for analysis [22]. Two phases of coding took place and a codebook was constructed. The initial phase involved an open-coding process, where the focus group facilitator and the observer used the focus group transcripts and their own notes to identify themes. A third research member who did not take part in the focus group also reviewed the transcript, then the team discussed similarities in the set of identified themes. The second phase of axial coding included comparing the themes across focus groups.

Faculty interviews were also audio recorded and transcribed verbatim for the analysis. Inductive-deductive qualitative analysis was used to discover themes in the responses, and predetermined themes mapped to the readiness framework were also sought . Each transcript was coded and new themes were added to the codebook as they emerged. By doing constant comparisons, research members were able to differentiate between similar themes and identify their relevant dimensions. With each addition of new data, themes were added and modified as needed. Finally, the resultant themes were combined to produce a coherent textural description of the phenomenon.

Data analysis and its verification were done independently by two researcher members. They read through the transcripts, identified the common themes separately then came together to discuss the results and reached an agreement regarding the themes and categories.

## Results

We conducted two focus group discussions that involved 4 fifth-year and 7 fourth-year students of CMED (N = 11) who aged between 21 and 23 years old, and 5 semi-structured interviews with 5 CMED clinical faculty members. The major themes elicited from the analyses explained the perceptions about the university’s decision and its communication to students, the perceived lack of clinical experience, students’ role as members of the medical team facing the pandemic, student safety, quality and design of VC and the skills it offered, belief in own ability to succeed in the VC, confidence that VC would reach its goals, new enhanced learning approaches, preparing students for new types of practice in the future, acquired skills, academic support and communication with faculty and college, and psychological support. (See Table 1 representing the themes and number of participants who expressed them).

**Table 1:** Themes and the number of participants who expressed them

Theme	Number of Students (N = 11)	Number of Faculty Members (N = 5)
***Discrepancy: understanding the need for the shift to VC*** Theme 1: Perceptions about the university decision and its communication to students	7 (63.64%)	2 (40%)
Theme 2: A perceived lack of clinical experience	9 (81.8%)	5 (100%)
***Understanding the necessity of the transition*** Theme 3: Students’ role as members of the medical team fighting the pandemic	3 (27.27%)	1 (20%)
Theme 4: Student safety	2 (18.18%)	3 (60%)
***Appropriateness*** Theme 5: Quality and design of VC and the skills it offered	5 (45.45%)	2 (40%)
***Efficacy*** Theme 6: Belief in own ability to succeed in the VC	1 (9%)	Not applicable
Theme 7: Confidence that VC would reach its goals	1 (9%)	2 (40%)
***Valence*** Theme 8: New enhanced learning approaches	9 (81.8%)	2 (40%)
Theme 9: Preparing students for new types of practice in the future	4 (36.36%)	2 (40%)
Theme 10: Acquired skills	2 (18.18%)	1 (20%)
***Principal Support*** Theme 11: Academic support and communication with faculty and college	6 (54.55%)	2 (40%)
Theme 12: Psychological support to students	2 (18.18%)	2 (40%)

### Discrepancy (understanding the need for the shift to VC)

#### Theme 1: perceptions about the university decision and its communication to students

Students (N = 7) were generally satisfied with the college decision to shift to VCs. To one of them, the college response was adequate, and she expressed, “College responded well when online learning was implemented the second week of the lockdown” [Student 2, year5, female]. Nevertheless, they were only partially satisfied in the way this decision was communicated to them, and with the slow response from college upon the start of the outbreak. One student mentioned,

“We went to the hospital, we did everything and we didn’t hear anything from the college. They only emailed us when we complained. Okay! I’m not sure if we’re supposed to complain every time just to get a response.” [Student 10, year4, male]

Faculty (N = 2) perceived a similar view as one of them expressed,

“During the early phase of the shift, no clear communication with students took place regarding how the clerkships will be handled. So none of us was ready. Neither the clinicians nor the patients and nor the students so all of them were not prepared for this. It just happened, you know, suddenly.” [Clinical Faculty 5]

However, all of the faculty members agreed that the college decision to shift to VC was appropriate, quick and reasonable.

“The plan, which has been put to let the students to join again, even for a shorter period of time starting from August for grade-4 and July for year-5, is a reasonable plan and it goes with the international, you know, its a fluidy situation

#### Theme 2: a perceived lack of clinical experience

Students (N = 9) were disappointed with the sudden change in learning, and felt it will be less beneficial than being in hospital. They realized a huge difference in the way clerkship was being provided, with a total “backward” shift from practice to theory.

“I personally realized a huge part of experience and learning was taken away and with no alternative at the moment. Because at the moment, we’re only taking online lectures” [Student 3, year4, female]

“taking us away from the clinical environment it was like taking us a step back to the college………we just got used to the way of history-taking and physical examination. Basically, our serious education was interrupted at that time.” [Student 5, year4, female]

More importantly, the vast majority of students emphasized the need for a quick return back to clinical sites. Some students said they were volunteering to compensate for lack of clinical exposure: “I volunteered for the emergency. Therefore, the emergency department volunteering has been really good “, a student expressed.

“We really need to go back, even though it’s risky… we would benefit more if we actually go back to the hospital or, for example volunteer or something during this situation. It would be, it would offer a great experience.” [Student 5, year4, female]

Students also tried to find other solutions by suggesting ways to communicate with patients online. Other Ideas included online consultations, WebEx meetings with patients and small training groups.

“We could have WebEx meetings with patients. Yeah, for example, like doctors can find a good case, they can communicate with the patient, ask him if he wants to participate with us for example and we can see the real patient, we can take history from him”. [Student 10, year4, male]

All the interviewed clinical faculty members echoed these views.

“I think we cannot replace it by virtual training. It might be in the future.. This is a stimulus in the future. People can do it, but again, it’s called practice of medicine. We cannot give it by books.” [Clinical Faculty 3]

### Understanding the necessity of the transition

#### Theme 3: students’ role as members of the medical team fighting the pandemic

Students (N = 3) perceived that they could have helped out in the pandemic and that would have been a major source of motivation for learning.

“When we used to go to the hospital, you feel motivated when you take care of the patient and you are a part of the team, whereas when you’re home and you just have a lecture.” [Student 1, year5, female]

”I think we could have helped and learned a lot if we were in the clinical situation.” [Student 4, year5, female]

A clinical faculty stated that students would better be practicing in hospitals because this would be their duty as future doctors during a pandemic.

“…. how to use The PPE [personal protection equipment], how to limit the chance of getting the infection once you’re a doctor, you cannot say no sorry I got kids. Well, everybody got kids. It’s our job. We are like soldiers and you cannot say, no I can’t fight the enemy because I got kids. [Clinical Faculty 4]

#### Theme 4: student safety

Only a few students (N = 2) had positive perceptions towards the VC and felt this move was to keep them and their families safe.

“As well thinking of my family as well. Not everyone has the same family situation that would allow them to actually go to the hospital with the outbreak there and go back home & make sure that everyone there is safe as well.” [Student 5, year4, female]

One of them also explained that he was expecting that even in the real setting, education would be affected, as the faculty will be too busy dealing with the pandemic.

“… but we need to look at the bigger picture. Okay! Like, the doctors who are taking care of us in the hospital are actually volunteering or concentrating on the COVID situation..., so we won’t have enough staff”. [Student 10, year4, male]

Contrarily, several students perceived that his move to VC might not be necessary because the students could have applied the infection control measures that they got extensively taught, and hence avoided losing the practical training.

“So I feel like, we could have taken the infection control measures that every other doctor, every other nurse, or every other hospital personal could have taken, and we could have avoided getting the infection if we took preventative measures and practiced good practice good hygiene in the hospital.” [Student 4, year5, female]

Clinical faculty members gave more importance to the issue of maintaining student safety, than the students themselves did. The majority (N = 3) agreed that moving to VC was the best decision to protect students and patients, and to avoid overwhelming the healthcare system.

“It’s, it’s the best why it’s the best for their safety is the best for the health care sector as a whole because of the need the need and the demand of the PPEs and for the patients as well.” [Clinical Faculty 5]

Other faculty members argued that instead of a total shift to VC, students should better maintain at least some limited exposure to clinical settings during the pandemic.

“So, it’s a bit difficult situation, but I’m still believing even limited clinical exposure was good idea even in the presence of the epidemic.” [Clinical Faculty 4]

Yet, some clinical faculty emphasized a need to provide students with more understanding of why such a shift is needed in order to make them embrace such a major change in their learning.

“they are still young and that is the point that we need to address to make sure that students know exactly why we do this new format and make sure that they’re always objective.” [Clinical Faculty 1]

### Appropriateness

#### Theme 5: quality and design of VC and the skills it offered

Almost half of students (N = 5) explained that they benefitted less from this clerkship compared to being in the hospital, as it did not provide them with the essential clinical skills. One student expressed, “Online lectures, not as useful as hands-on training and the lectures don’t work for me… since the pandemic started, I didn’t really gain any clinical experience.” All students agreed that the VC was mainly theory and knowledge-based, and could never compensate for the actual clinical placement, which is an integral part of their learning.

“we need to communicate with the patients, take the history ourselves. after we do the physical [physical examination], we can go discuss it with a supervisor and the doctor will comment on what we did, this kind of interaction you cannot replace it with online lectures”. [Student 10, year4, male]

Students negatively perceived the design of the VC. They felt that the VC was less appropriate than on-site training because doctors were busy and were not able to deliver education that optimally matched with the university’s program requirements and one of them expressed, “Doctors are busy, and some have a gap in education based on the college blueprint [the set of educational outcomes].”

Students believed the low number of patients seen during the pandemic deeded a compensation mechanism, such as frequent formative assessment.

“Surgery was the only rotation that I did not observe any patient in it. I feel if we take assessment. Daily assessment, regarding each topic in order to fulfil all the gaps regarding surgery, for example for us people who did not attend their rotation”. [Student 2, year5, female]

Students who were through their first semester in clerkships had to go through medical rotations for ten weeks and a surgical rotation for a similar period. The disruption coincided with the beginning of the second rotation and hence affected the introduction to those rotations. That was perceived by some students as lack of clear goals for the clerkship

“The first four weeks we were lost, because we don’t know what is E-value [a web-based platform used for scheduling and student assessment] we don’t know what we’re expected to do. We don’t know what are the goals!” [Student 6, year4, male].

On the other hand, students opinions regarding the extent of interaction and engagement during the online sessions were variable. Some students felt that online lectures were missing the interactive component. However, others valued the clinical faculty exerted efforts towards overcoming this issue. They explained how faculty applied several techniques to enhance the appropriateness of the VC by making the lectures more interactive and engaging.

“After the lecture, some doctors used to put a lot of questions, like, more than ten questions after the lecture to summarize the points that they’re raising…. Doctors made us all join with the camera and the Mic, and also he [faculty member] was asking us to participate by the name. So, in this way, it was more engaging”. [Student 2, year5, female]

Clinical faculty members’ stressed on the importance of students physical presence in clinical settings and that it is irreplaceable.

“I think this time of training, they need to see patients themselves, they need to put their hands on the patients. And this honestly has been jeopardized...…Again, I think they need to start from the way they sit in that clinic from the way the sit on the chair, introduce themselves to their patients…... It’s very difficult. Because if they read it, they will forget it. It’s a practice. They should do it when they sit in the clinic…” [Clinical faculty3]

To faculty, the VC created a need to re-structure the standard tests of clinical skills and the Objective Structured Clinical Examination (OSCE) mode of assessment. They expressed that different learning outcomes were expected from different rotations, and such specific outcomes and objectives should have been better clarified to students from the beginning in order for them to make realistic expectations from the VC.

“In the clinical clerkship, we did the structured case discussion. It went fine, but our expectation did not match what the student have and I think is the major thing, because we cannot, based on assessment, without knowing exactly what the students have gained through their clinical training…, there should be a clear objective and by the end you achieve it.” [Clinical Faculty 1]

### Efficacy

#### Theme 6: belief in own ability to succeed in the VC

Only one student felt confident that students will succeed in this move to VC and expressed, “Being the first batch we’ve been through so many changes and we adapted to them… I have faith in the college”. However, the majority of students felt discouraged and unsatisfied with the lack of hands-on-training and one of them explained, “Online lectures, not as useful as hands-on training and the lectures don’t work for me”. Another student added, “It is a little bit discouraging at times, so I find it a struggle to get back to reading things without knowing I’ll ever see those things or experience them in the hospital. “.

#### Theme 7: confidence that VC would reach its goals

The vast majority of students felt that the lack of gaining clinical skills along with the lack of staff availability and lack of organization during the pandemic would not help the program achieve its goals.

“making the doctors and the faculty aware that we have a program and these are the goals one, two, three, and these are the expected learning outcomes and the students need to perform one, two, three, four and we may receive assistance from the doctors and the hospital administration for that we are able to do a few things, but in a lot of circumstances, we lose that chance.” [Student 6, year4, male]

Conversely, faculty (N = 2) held more positive views about students’ efficacy towards taking the VC. They mentioned how a condensed training program was given to students, at least for some rotations (such as the pediatrics rotation), which was an efficient way to make the VC achieve its goals.

“I am confident in what they [students] had in terms of information by the end of the rotation and actually, see the difference between the first week of rotation and the last week of the rotation and it shows while they are doing the oral presentations” [Clinical Faculty 2]

Faculty also had confidence in student’s abilities to carry the VC based on their positive attitude and attributes. According to them, students built the needed knowledge, were motivated to learn and were highly adaptable.

…The attitude is very important that I think because we could make sure that students are motivated enough, and they are working towards and proceeding their rotation, make sure they have a clear objective”. [Clinical Faculty 1]

“Overall. They were good. Some of them were very enthusiastic about learning …They are confident, they are very good”. [Clinical Faculty 2]

They also valued how this millennial generation of students accommodated the shift to virtual learning, since they possessed high computer and technological skills.

“I think the students also, I was astonished, the way the absorb the... how they understood the system and they had no problem about the online teaching at all” HCP4

### Valence

#### Theme 8: new enhanced learning approaches

Almost all students (N = 9) appreciated some short-term benefits from VC that were not feasible in the context of on-site trainings. They explained how they were offered extra sessions and revisions and how the clinical staff were always approachable to answer questions over the phone. It is worth indicating that attempts to standardize clinical lecture across different remote clinical sites wasn’t possible due to the lack of IT infrastructure in place. That lead to the same lectures prepared and provided by different faculty each in their own hospital which was always a source of students dissatisfaction before the pandemic. Once these lectures moved online during the pandemic, students started receiving each lecture by a single faculty regardless of the location of their clinical placement, which they appreciated and considered as an enhancement in learning approaches in addition to the fact that faculty started including case-based learning in some online sessions. One of the students mentioned:

“The online lectures. It’s much better compared to the lectures that we take in hospital… I feel that our lectures in hospital can be replaced with online lectures to be much better even if we can take it evenings, or even at night”. [Student 9, year4, male]

”Doctors would give us the lecture then give us like, cases and we resolve questions. So, it’s kind of like seeing a patient and discussing.” [Student 1, year5, female]

The majority of students also agreed that the VC helped them allocate more time for studying and revising patient cases.

“.. It was really difficult to study after a whole day of the training, which is from seven to three. You are very tired, you’re not motivated to study or do anything for five days, it’s only the weekend when you have the opportunity to the study or to revise what you saw at the hospital. ” [Student 9, year4, male]

Faculty members’ views coincided with views of students.

“we believe now that we can do a lot of things without presence physically. And one of the things that you are doing your job now, even from other places, I think we become more accessible to our students by this telecommunications…” [Clinical Faculty 3]

#### Theme 9: preparing students for new types of practice in the future

Students (N = 4) and faculty members (N = 2) perceived that the VC as an opportunity with long-term benefits for students, such as preparing them for virtual teaching and practicing telehealth in the future.

“…. the online teaching, there will be a, it has a lot of future. So, they can use it in the future themselves to teach somebody else we can give lectures online.” [Clinical Faculty 4]

“I think this will be a major change in the healthcare system, the appointments and the frequency of the appointments, and having the virtual evening clinics will be a great change”. [Clinical Facutly 5]

#### Theme 10: acquired skills

During the VC, students (N = 2) developed some skills like adaptability and problem-solving, which are very important skills.

“They teach us how to adapt to situations quickly even the college everyone had to adapt to the situation thrown at them very quickly, find solutions to every problem. So, everyone had to look, think quickly and change”. [Student 8, year4, male]

### Principal support

#### Theme 11: academic support and communication with faculty and college

Students (N = 6) were satisfied with the positive attitude of faculty, their availability and willingness to help at any time. “The [clerkship] director is always approachable”, one student described. “The attitude of the professors and of the doctors have been really good. They’ve been really friendly. They’re really keen on teaching us”, another student explained.

“I feel like we had a lot of support from them. The staff are willing to give extra sessions and revision sessions and willing to take questions at any time. They have given us their contact numbers as well.” [Student 4, year5, female]

On the other hand, a few students felt uncomfortable with the level of communication and they asked for clearer and more frequent communication about academic issues from the college. These students asked for better organization and more collaboration within the program in a way that facilitates better and efficient communication regarding assessments, curriculum and resources available during the VC. One student explained how this would also allow more depth while studying a specific topic.

“So when I’m studying, I want to know how much in depth should I go so I can read a sufficient amount on it. If you say abdominal pain, there could be a lot of topics under that. So having a specific depth you want….that’s the issue I’m facing with the blueprints at the moment”. [Student 8, year4, male]

Students also expressed that they did not receive sufficient guidance nor regular feedback on their performance and thus demanded more frequent assessments. They also asked for more support from college by assigning staff from the college as student mentors.

“We need motivation, guidance and kind of follow up, which is actually the role of mentors… So, if we had, for example, mentors from the college, this would be much better.” [Student 9, year4, male]

Faculty shared the same positive views as the students regarding the easier access to faculty members during the VC.

“…all what you can do now is actually a plan easy to access to faculty. Students have the right to ask questions, find their faculty, even if we have answers for extra time” [Clinical Faculty1]

In concordance with students’ views, clinical faculty members’ demanded more administrative support from college. Clinical faculty members indicated that some disorganization and insufficient communication existed, and were mainly caused by the lack of administrative and technical support from training sites.

“We do need admin support, because I can describe how much we struggled with that at Sidra/ Hamad. Because I am working with two groups here and there, it is like, I do not know, print the schedule. I like to print the schedule sitting in my office. So I can allocate which student is rotating where or whatever phone I get. I know where they are. We have beautiful schedules made by XXXX colorful and last month, when I asked the nurse to print this paper for me, she said no colored printers.” [Clinical Faculty2]

“I think quite possibly build the station in the medical education where the faculty can go to, full prepared studio, good mike for example, with more advanced computers in order to make sure that we deliver the best possible medical education.” [Clinical Faculty1]

#### Theme 12: psychological support to students

Faculty members (N = 2) perceived a need to provide students with more specialized psychological support to lessen the anxiety that student might have in relation to the pandemic and its impact on their education. They indicated that faculty communicated well with students about this issue. The college also assigned a student representative board, but no student counsellors, to work closely with students, to help them understand the need to change the nature of their trainings.

“Having these representatives of students, provided psychological support to the students that we are listening to. We are there… having a very close communication between the students and the college at this critical situation… the more the communication, the more students have trust and confidence towards the faculty and the college…” [Clinical Faculty 5]

“I would suggest, in terms of coping strategies and coping mechanisms, that the university provides a counselor on the phone, or WebEx, or some hotline...not because of the COVID alone, but to this age group.” [Clinical Faculty2]

## Discussion

The primary objective of this study was to explore the perceived readiness and experiences of CMED student at QU towards shifting to full scale VC during the COVID-19 pandemic. By applying the theoretical framework of readiness for change, we were able to find that CMED students were not fully ready for the shift to VCs, and held mixed perceptions towards such a change.

The majority of CMED students (81.8%) and all faculty members who participated in this study believed that the VC could never compensate for the lost clinical experience, which are normally the most important aim of clinical rotations. Such a finding is consistent with the literature about the importance of interaction with patients and other healthcare providers, during clinical trainings as the cornerstone for shaping up scientifically grounded and clinically competent physicians [23]. In spite of the several advantages of videoconferencing technology used in medical education, literature suggests that it can constrain dialogue and does not allow for teaching psychomotor skills [5], and students may struggle with clinical skills later [5, 13].

Results from this study show that medical students (63.64%) experienced a high sense of uncertainty during the shift to VC in such an emergent situation, and that students expected clearer and more prompt communication from the college about their decisions and the changes they applied.

Students (45.45%) also perceived that shifting to VC was not appropriate. Most of the students felt that having a theory-based training instead of an experiential one was discouraging. They were disappointed with not being offered any hands-on experience during the VC, as the latter could only be gained through collaboration and peer-review [23, 24]. These perceptions coincided well with what students reported in similar studies done in other parts of the world [10, 14, 25–28].

Consequently, a few students (9%)were skeptical about their own ability to succeed in the VC, and whether the VC would reach its experiential learning goals. For example, medical sstudents felt uncomfortable with the level of organization and collaboration across departments. They asked for clearer and more frequent communication from the college in order to allow for more efficient planning of their studying schedule. Insufficient communication with educators can be a major obstacle to building confidence and capability to undertake new challenges and changes [29–33]. However, clinical faculty felt that students had great ability to navigate through their virtual learning experience based on their adaptability and willingness to learn. They also possessed high technological skills. This result is similar to findings those of another study from Nepal [14] but discordant with results from 2 others studies in KSA [10, 11].

A major finding from our study was the students’ motivation to remain on-site. Although the study was conducted around the time where the pandemic was at its peak, personal safety at training sites was not much of a concern to most of the students (18.18%). These students believed they could have applied the infection control and safety measures while maintaining their training. Moreover, they (27.27%) believed that, being future medical professionals, taking part in the fight against the pandemic was a professional obligation. They were disappointed with not being part of the medical team and demanded an urgent need to return to training sites. Literature has demonstrated various student views in this regard. Our findings are concordant with those from Singapore [34], which assessed the medical students’ preference and showed higher preference for returning to the clinical setting during the COVID-19 pandemic, and attributed this preference to a high level of internal motivation, professional responsibility of students and the little fear that they would pose risk to patients and to the healthcare system [34]. From a similar perspective, some institutions were concerned about the type of message that moving to VC would send to medical students regarding a medical doctor’s obligation to treat all patients [35, 36]. Furthermore, other medical schools recruited students, medical residents and promoted final year medical students to clinical service in order meet the high demand of doctors during the pandemic [36–38]. Looking back at literature in 2003, during the height of the SARS outbreak in Singapore, we can see that medical students were assigned to help with temperature screenings [39]. Student perceptions regarding onsite training in our study were different compared to findings in other studies [40, 41]. For example, and in contrary to what students in our study expressed, the majority of medical students in Brazil had negative perceptions towards being at training sites during the pandemic and many medical schools prioritized maintaining student safety, patient safety and preservation of Personal Protective Equipment (PPE) [41]. Another study called for a need for paradigm shift in medical education traditions and curriculum, to empower students and make them ready to face emergent health crisis, and concluded that no further expectations should be set for students until such measures become in place [42].

The most positive student perceptions in this study were expressed towards the short term and the long-term benefits that they gained from this VC experience. There was an agreement among students (81.8%) that the VC offered several new enhanced learning approaches. They expressed high satisfaction with the case-based learning that was offered to them, and valued the opportunity they had by being introduced to telehealth, which constitutes a very important asset to their future practice [5, 43]. Students also appreciated being able to allocate more time for studying and revising patient cases. They also developed some skills like adaptability and problem solving. Such student perceptions are consistent with what was reported in KSA [10, 28].

Students (54.55%) were generally satisfied with the positive attitude and academic support of faculty members in terms of their availability and willingness to help students during the VC. Nevertheless, students were not satisfied with the extent of college communication, guidance and feedback. They also demanded more support from the college by assigning mentors, and asked for more efficient feedback on their performance by having more frequent and timely formative assessments. For instance, a systematic review by Peechapol et al. consistently listed instructor feedback among the main factors that influence students’ self-efficacy in online learning [31, 35].

Additionally, students reported that they received negligible psychological support from the college during the VC. A student representative board, but no student counsellors, were assigned by the college to work closely with students in order provide psychological support and lessen the apprehension they had regarding the impact that the pandemic would have on their education. There is a clear evidence from literature that medical students are subject to academic stress which in turn makes them at risk of poor mental health status and eventually suicidal ideation [44, 45]. This also goes along with existing literature regarding a deep need for improving medical students’ preparedness in order for them to face challenges and threats imposed by COVID-19 on their education, as well as by the health consequences of the pandemic itself [42].

Findings of this study may be limited to participant’s interpretation of experiences and perceptions may have been subject to researcher’s personal biases [46]. Results from this study may not reflect the views and experiences of students and clinical faculty members of other medical schools in Qatar. No validation of the themes by study participants was done due to time constraints.

## Conclusion

This is the first study in the Arab region to ever explore medical students’ readiness to shift to full scale VC during the COVID-19 pandemic. Student views reflected how they perceived themselves as more than just learners, and that they were willing to serve some value-added roles in the provision of care during an emerging health crisis. Hence, it would be hence important to include them in the decision making and ensure proper communication with them about the need to shift to VC and about future plans for better incorporation of such major changes in their learning [35]. Efforts should be made in order to ensure medical students’ safety and that of the patients without compromising the quality of clinical trainings and recognizing potential contributions that medical students can offer during a health crisis. Thus, more focus should also be placed on improving medical student preparedness as one mediator for their readiness to undertake VC [42]. Medical curricula need to create channels to accommodate more in-depth learning approaches in the presence of the COVID-19 pandemic and other emegencies. Therefore, it would be beneficial to assist remote learning by virtual simulations and computer-based models [5, 6, 10, 43, 47]. Finally, this study can aid in understanding medical students’ current experiences in order to prepare competent medical doctors who will essentially advance healthcare outcomes and fulfill the healthcare needs of Qatar.

## Electronic supplementary material

Below is the link to the electronic supplementary material.

Supplementary Material 1

## Data Availability

Available from the corresponding author upon request.

## Abbreviations

QU: Qatar University
QU Health: Qatar University Health
VC: Virtual Clerkship
MD: Doctor of Medicine
CMED: College of Medicine
OSCE: Objective-Structured Clinical Examination
PPE: Personal Protective Equipment
KSA: Kingdom of Saudi Arabia
