# Correction to: A qualitative assessment of medical students’ readiness for virtual clerkships at a Qatari university during the COVID-19 pandemic

**DOI:** 10.1186/s12909-023-04403-0

**Published:** 2023-06-20

**Authors:** Hiba Bawadi, Ayad Al-Moslih, Rula Shami, Xiangyun Du, Alla El-Awaisi, Hanan Abdul Rahim, Ghadir Fakhri Al-Jayyousi

**Affiliations:** 1 Section Head of Clinical Education, Vice President for Medical and Health Sciences Office, QU Health, Qatar University, PO Box 2713, Doha, Qatar; 2 Section Head of Pre-Clinical Education, College of Medicine, QU Health, Qatar University, PO Box 2713, Doha, Qatar; 3 College of Health Sciences, QU Health, Qatar University, PO Box 2713, Doha, Qatar; 4 College of Education, Qatar University, PO Box 2713, Doha, Qatar; 5 College of Pharmacy, QU Health, Qatar University, PO Box 2713, Doha, Qatar

Correction: BMC Medical Education (2023) 23:186 10.1186/s12909-023-04117-3

Following publication of the original article [1], due to a typesetting error, references [19] and [20] were written incorrectly. The correct references are given below:

[19] Bawadi H, Abdul Rahim H, Moawad J, Shami R, Du X, El-Awaisi A, Al-Moslih AMI, Diab M, Al-Jayyousi GF. Health sciences students’ and instructors’ perceptions of the emergency switch to virtual internship amid the COVID-19 pandemic: A case from Qatar. Front Med (Lausanne). 2022 Aug 9;9:939416. doi: 10.3389/fmed.2022.939416. PMID: 36,059,828; PMCID: PMC9435433.

[20] Bawadi H, Shami R, El-Awaisi A, Al-Moslih A, Abdul Rahim H, Du X, Moawad J, Al-Jayyousi GF. Exploring the challenges of virtual internships during the COVID-19 pandemic and their potential influence on the professional identity of health professions students: A view from Qatar University. Front Med (Lausanne). 2023 Jan 30;10:1107693. doi: 10.3389/fmed.2023.1107693. PMID: 36,793,877; PMCID: PMC9922901.

Also, in the Conclusion section, there is a spelling mistake. The incorrect and correct version is highlighted in **bold typeface**.

Incorrect:

Medical curricula need to create channels to accommodate more in-depth learning approaches in the presence of the COVID-19 pandemic and other **emegencies.**

Correct:

In the conclusion, there is a spelling mistake:

Medical curricula need to create channels to accommodate more in-depth learning approaches in the presence of the COVID-19 pandemic and other **emergencies.**

The publishers apologize for this error.

The original article has been corrected.
