# Supplementary material for: A qualitative assessment of medical students’ readiness for virtual clerkships at a Qatari university during the COVID-19 pandemic
Source: BMC Med Educ. 2023 Mar 27;23:186. doi: 10.1186/s12909-023-04117-3 (PMC10042106; doi:10.1186/s12909-023-04117-3)
Supplement: Supplementary file 1 — Supplementary Material 1 [file 12909_2023_4117_MOESM1_ESM.docx]

Appendix-1: Focus group questions for students

1. **Describe your current experience of the clinical training, please.**

Follow up question (after they share thoughts):

Ask about their daily practices and time management/schedules to get more details.

e.g. Can you describe your current routine? How is a day like for you?

1. **How appropriate do you find the current design of training?**

Probes, if students didn’t respond well following the above:

What skills/attitudes/knowledge do you think you need to succeed in this virtual clerkship program?

Do you feel that you have those skills/attitudes/knowledge right now? (probe: why/why not)

(appropriateness)

1. **What support or preparation you think you need to succeed in reaching the goals of the program?**

Do you currently feel supported, by clerkship directors, college, preceptors, or instructors?

(leadership support)

1. **In which way do you think the program will support the success of reaching the expected goals?**

In which ways it may not?

(discrepancy)

1. **How confident do you feel to go through the training program and reach the expected goals?**

In which ways you feel your instructors/mentors and the overall program share this confidence?

(efficacy)

1. **How do you see these experiences benefit your career and life in the long run?**

What in this program prepares you the most for your future role?

What in this program prepares you the least for your future role?

What part of your future role are you most/least confident about after completing this program?

(Valence)

1. **How would you assess your program’s response to the current COVID19 situation?**

(if the answer is general, probe to the specific handling of the clinical training).

1. **What are the challenges from your point of view?**

Can you please elaborate with examples? What do you do to overcome these challenges?

(reaction and action)

1. **What are the good practices that you experienced so far?**
2. **What suggestions would you provide to improve your training?**

Appendix-2: Interview questions for clinical faculty/preceptors

Q1. Can you please describe the current version of the clinical training you are looking after? What are the major differences compared to the face-to-face version?

Q2. In which ways you think students were ready for this shift e.g. virtual education/training? (how prepared do you think were the students?)

Q3. In general, what skills/ attitudes/ knowledge do you think are needed for a student to successfully navigate through their program?

Q4. How did students obtain the needed understanding, knowledge and skills for this program?

Q5. What kind of support is needed and in what way is it currently provided by yourself and the program?

Q6. How confident are students in their online skills?

How confident do you find the students about the overall program design and in the preceptors and so on?

Q7. How confident do you feel bout the current shape of the program?

Q8. In which ways you think students believe this training experience would benefit their future career?

Q9. How do you perceive the challenges students have encountered in this clinical training experience in the current overall situation from a preceptor’s perspective?

- Can you please elaborate with examples?
- What do you do to overcome these challenges?

Q10. How do students cope with the challenges?

Q11. What are the potential benefits from your perspective for both students and preceptors?

Q12. What suggestions would you provide to improve the current situation of the program?

Further questions asked to seek further elaboration:

Q13. How did assessment of students change with the shift in educational approaches during the pandemic?

Q14. How do you imagine the clinical training in the post-pandemic period?
